# Theoretical Studies for the Discovery of Potential Sucrase-Isomaltase Inhibitors from Maize Silk Phytochemicals: An Approach to Treatment of Type 2 Diabetes

**DOI:** 10.3390/molecules28196778

**Published:** 2023-09-23

**Authors:** Linda-Lucila Landeros-Martínez, Mara Ibeth Campos-Almazán, Nora-Aydeé Sánchez-Bojorge, Raul Flores, Juan Pedro Palomares-Báez, Luz María Rodríguez-Valdez

**Affiliations:** Facultad de Ciencias Químicas, Universidad Autónoma de Chihuahua, Chihuahua 31125, Mexico; lilanderos@uach.mx (L.-L.L.-M.); ialmazan@uach.mx (M.I.C.-A.); nsanchez@uach.mx (N.-A.S.-B.); raul_flores@comunidad.unam.mx (R.F.); ppalomares@uach.mx (J.P.P.-B.)

**Keywords:** acarbose, maysin, luteolin, sucrose-isomaltase, molecular docking, molecular dynamics, quantum chemical

## Abstract

A theoretical analysis of the potential inhibition of human sucrase-isomaltase (SI) by flavonoids was carried out with the aim of identifying potential candidates for an alternative treatment of type 2 diabetes. Two compounds from maize silks, maysin and luteolin, were selected to be studied with the structure-based density functional theory (DFT), molecular docking (MDock), and molecular dynamics (MD) approaches. The docking score and MD simulations suggested that the compounds maysin and luteolin presented higher binding affinities in N-terminal sucrase-isomaltase (NtSI) than in C-terminal sucrase-isomaltase (CtSI). The reactivity parameters, such as chemical hardness (η) and chemical potential (µ), of the ligands, as well as of the active site amino acids of the NtSI, were calculated by the meta-GGA M06 functional in combination with the 6-31G(d) basis set. The lower value of chemical hardness calculated for the maysin molecule indicated that this might interact more easily with the active site of NtSI, in comparison with the values of the acarbose and luteolin structures. Additionally, a possible oxidative process was proposed through the quantum chemical calculations of the electronic charge transfer values (∆N) between the active site amino acids of the NtSI and the ligands. In addition, maysin displayed a higher ability to generate more oxidative damage in the NtSI active site. Our results suggest that maysin and luteolin can be used to develop novel α-glucosidase inhibitors via NtSI inhibition.

## 1. Introduction

Type 2 diabetes mellitus is a chronic disease that encompasses a group of complex metabolic abnormalities. These abnormalities include high blood glucose levels [1], poor insulin secretion by pancreatic beta cells [2], and an ineffective response of cells to insulin [3]. For many years, acarbose (ACA) has been used as a treatment for this condition. ACA is a pseudotetrasaccharide that competitively and reversibly inhibits intestinal α-glucosidases, which are enzymes involved in the digestion of complex polysaccharides and sucrase [4,5]. Two types of human intestinal α-glucosidase, maltase-glucoamylase (MGAM) and sucrase-isomaltase (SI), have two duplicated catalytic domains: the N-terminal (NtMGAM, NtSI) and C-terminal (CtMGAM, CtSI) [6]. The N-terminal domain of the isomaltase subunit anchors the protein to the cell membrane, while the C-terminal domain is located at the luminal. Both domains show a sequence homology [7]. These α-glucosidases play important roles in the release of glucose monomers from postamylase starch products [8].

However, ACA presents several adverse effects, such as flatulence, abdominal distension, borborygmus, diarrhea, and so on [9]. The use of natural options has emerged as a therapeutic alternative for the treatment of diseases like diabetes. Natural products have demonstrated inhibitory effects on the MGAM and SI [10,11,12]. In this regard, previous studies have reported that maize silk stigma extracts can inhibit α-glucosidases [10,12]. These extracts are known to contain a mixture of sugars and polyphenols. In a previous investigation, our research group evaluated in vitro the hydroalcoholic extract from maize silks, characterized by a high polyphenolic compounds content, showing its capacity to inhibit the catalytic activity of crude intestinal α-glucosidases extract [10]. Furthermore, an in silico analysis suggested that MAY, a component of the hydroalcoholic extract from maize silks, could be responsible for inhibiting Nt- MGAM (an α-glucosidase) [10,13]. The MAY compound’s structure consists of a luteolin (LUT) core attached to a rhamnosyl-6-C-(4-ketofucosyl) moiety. It has been reported that LUT itself acts as an inhibitor of α-glucosidases [14,15,16,17,18]. However, the precise mechanism underlying the biological activity of MAY and LUT, as investigated in the aforementioned study, on SI remains unclear. Therefore, a theoretical study examining the molecular interactions between these compounds is of great importance.

Currently, theoretical studies such as quantum chemical calculations, molecular docking (MDock), and molecular dynamics (MD) simulations play a crucial role in rational drug design [19]. Density functional theory (DFT) has been employed to study enzyme–inhibitor interactions in medicinal chemistry and drug design [20]. MDock is the most popular computational method for drug design as it predicts the preferred orientation of a small molecule in relation to a macromolecule when they bind to form a complex [21]. This method is often complemented by molecular dynamics (MD) simulations, which explore binding pathways of active molecules in solution to aid in the selection of successful candidates [19]. Several analyses employing MD have reported replacement sugars in insulin resistance [22] and the determination of binding mechanisms of glucoamylase inhibitors through an integrated modelling study combining MD simulations and binding free energy calculations [23], as well as MD analysis of α-amylases and intestinal glucosidases using ACA and miglitol [24].

Therefore, an analysis of MDock, MD, and quantum chemical calculations with ACA as a positive control was carried out in this paper. Additionally, MAY and LUT were studied to examine their potential inhibition on the SI domains, namely NtSI and CtSI. The results obtained in this work provide useful information that contributes to the understanding of inhibition of human SI by flavonoids. Furthermore, they offer insights into the chemical features and potential inhibition of MAY and LUT targeting this enzyme. The results obtained in this study will enable a detailed analysis of the possible mechanism of action of these compounds as an alternative treatment for type 2 diabetes.

## 2. Results

### 2.1. Structural Optimization of Ligands

**Figure 1:**
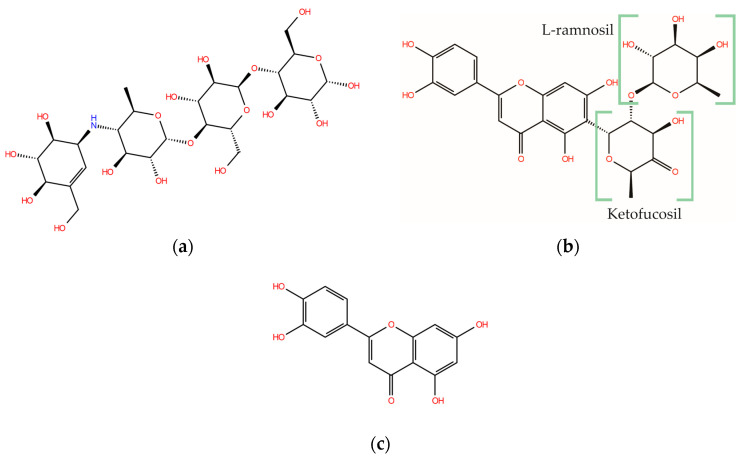
Structure of (**a**) ACA, (**b**) MAY, and (**c**) LUT.

The structures of the α-glucosidase inhibitor (ACA) and the polyphenolic compounds from maize silks (MAY and LUT) are shown in Figure 1. ACA is a pseudosaccharide (Figure 1a). MAY is a glycosylated flavonoid composed of a luteolin unit linked to ketofucosil and L-ramnosil (Figure 1b), where these latter two functional groups are key for the binding of MAY with the N-terminal subunit of SI, which will be described in detail later in the discussion section. Lastly, LUT is a tetrahydroxyflavone (Figure 1c).

**Figure 2:**
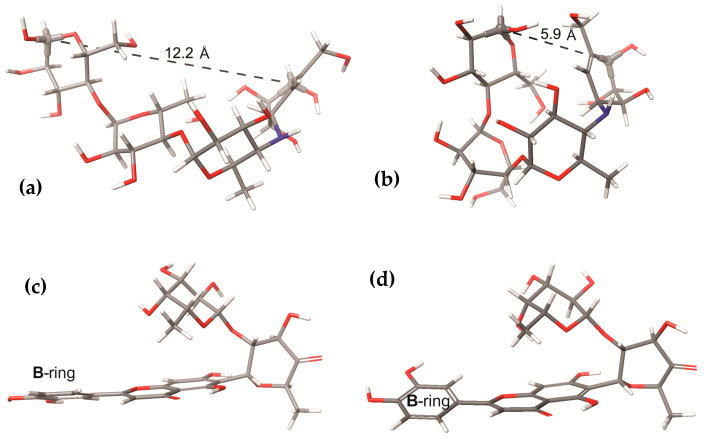
Global minimum structures of (**a**) ACA in water solution, (**b**) ACA in gas phase, (**c**) MAY in water solution, and (**d**) MAY in gas phase. Dashed line is the end-to-end measurement of ACA.

The rotational freedom degrees of MAY and ACA result in a large number of local minima. The search of the global minimum using the CREST tool located 11, 581, and 1604 conformers for LUT, MAY, and ACA, respectively. As expected, only a few LUT conformers were found due to its small size, with only the rotation of its hydroxyl groups. The global minimum conformation of ACA differs significantly between the gas phase and water solution, as shown in Figure 2a and b, respectively. This can be observed in the distance from side to side of the ACA structure, which measures 12.2 Å in water solution and 5.9 Å in the gas phase, highlighting the solvent effect on the ACA structure. The solvent effect is not significant for the MAY molecule (Figure 2c,d), where only small rotations are observed in the hydroxyl groups and the B-ring (polyphenolic moiety).

An optimization using DFT was performed on the selected conformers obtained from the CREST tool. These conformers were chosen based on a significant root mean square deviation (RMSD) (cut-off > 1 Å) from the global minimum for ACA and MAY. The relative energy values calculated in the selected conformers indicated that the global minimum found with the CREST tool in a water solution agrees with the DFT optimization using the M06 functional and the 6-31G(d) basis set employing the implicit solvent model SMD (See Table 1).

### 2.2. Molecular Docking Analysis between Ligands, ACA, MAY, and LUT, with the Sucrase-Isomaltase Domain B and D

**Figure A1:**
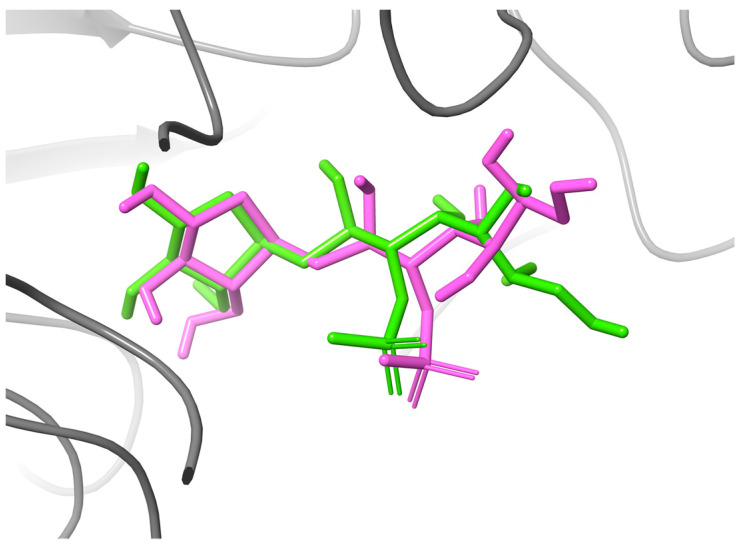
Comparison between the experimental binding mode (PDB: 3LPP, pink) and the top-scoring AutoDock Vina pose (green) for the compound kotalanol. The binding site SI is displayed as a gray ribbon.

On the other hand, the global minimum structures for ACA and MAY were subjected to MDock analysis in NtSI (domain B) and CtSI (domain D); as previously mentioned, domain B functions as the attachment point for the protein to the cell membrane, whereas domain D is orientated toward the lumen. In order to validate the molecular docking protocol carried out in the present study, a re-docking of the crystallized inhibitor kotalanol with the SI was performed (see Figure A1 in Appendix A section). According to the docking results, the kotalanol maintained a similar binding pose as that observed experimentally in crystal structure 3LPP. In addition, this inhibitor and the studied compounds in this work (See Table 2) shared interactions with the residues HIS629, ASP355, ARG555, ASP231, TRP435, PHE479, and TRP327. The docking score obtained from MDock analysis indicated that the compounds exhibited better binding energy values in NtSI compared to CtSI (Table 2). In addition, the residues that interacted with each domain were different. In NtSI domain B, ACA was in contact with 13 residues, TRP327, ASN328, LYS330, ILE356, TRP435, TRP470, ASP472, MET473, ARG555, ASP571, PHE604, HIS629, and ASP632. The binding site for MAY-NtSI was formed by the residues ASP231, LEU233, TRP327, ASP355, ILE356, TRP435, TRP470, ASP472, MET473, SER477, PHE479, LYS509, HIS629, and SER631. Finally, the binding site residues for the complex LUT-NtSI were ASP231, TRP327, ASP355, ILE392, TRP435, TRP470, ASP472, PHE479, LYS509, ASP571, PHE600, and SER631. For CtSI domain D, ACA interacted with 13 residues: ASN43, ILE45, PRO46, GLU47, GLN48, PHE49, PRO50, SER68, LEU69, THR224, PHE272, ARG282, and LYS594. The MAY complex with CtSI domain D was in contact with ASP231, GLN232, LEU233, TRP327, ASP355, ILE392, TRP435, TRP470, and LYS509. Lastly, the binding site for LUT in CtSI domain D consisted of the residues LEU311, ALA313, ARG549, ARG563, HIS629, and LYS805. On the other hand, the interactions established by ACA with the active site residues of each SI domain differed from those of LUT. Meanwhile, MAY and ACA exhibited a common interaction with TRP435 in Nt-SI, involving a π–π type interaction and a hydrogen bond, respectively. The molecular dynamics section will describe how some of the interactions obtained by molecular docking were preserved.

### 2.3. Molecular Dynamics Simulation Analysis

**Figure 3:**
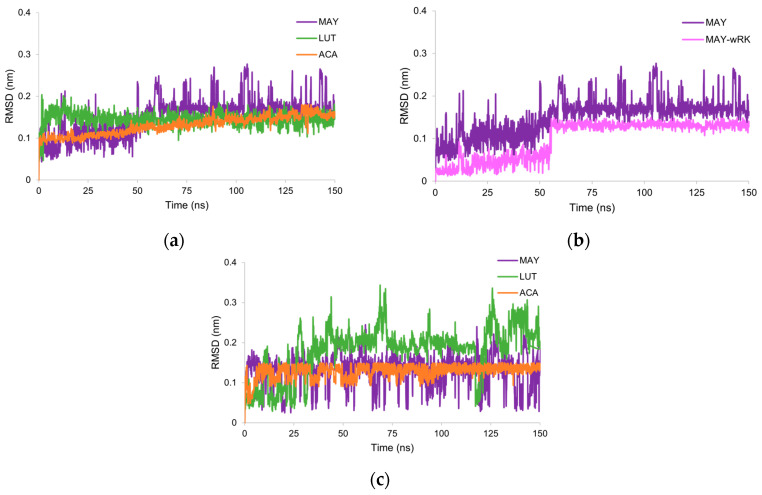
(**a**) RMSD values of the heavy atoms in the complexes of ACA, MAY, and LUT with NtSI domain B. (**b**) RMSD values of atoms of maysin (MAY) and atoms of polyphenolic moiety of maysin without rhamnosyl-6-C-(4-ketofucosyl) group (MAY-wRK). (**c**) RMSD values of atoms of the complexes of ACA, MAY, and LUT with CtSI domain D.

The RMSD values of each ligand (considering only heavy atoms) in complex with NtSI domain B and CtSI domain D during MD simulation were calculated to assess the stability of the ligands in the active site of these subunits (Figure 3). Most RMSD values reached a plateau after 50 ns production step, while the RMSD values for MAY continued to fluctuate (Figure 3a). Therefore, RMSD calculations for the MAY polyphenolic group were performed without considering the rhamnosyl-6-C-(4-ketofucosyl) moiety. Figure 3b shows that the polyphenolic moiety remained stable throughout the entire MD simulation, indicating that RMSD fluctuations observed for MAY were influenced by displacements of the rhamnosyl-6-C-(4-ketofucosyl) moiety. Conversely, both MAY and LUT did not maintain conformational stability in the CtSI (domain D) binding site compared to ACA (Figure 3c), with coefficients of variation of 31 and 35%, respectively. These results suggest that the binding site for MAY and LUT is likely located in the NtSI (domain B) active site. In addition, these results were consistent with the data obtained from molecular docking.

May and LUT had a more stable posture in domain B; hence, only NtSI (domain B) in complex with each ligand was analyzed. Molecular mechanics with Poisson–Boltzmann and surface area solvation (MM/PBSA) was used to predict binding free energies (Table 1A in Appendix A). Interestingly, MAY’s binding free energy was −14.09 Kcal/mol, similar to acarbose’s −15.52 Kcal/mol. Moreover, MAY has a better binding free energy (−14.09 Kcal/mol) than LUT (−9.32 Kcal/mol). MAY’s significant negative binding free energy suggests it may be a potential SI inhibitor. Since MAY and LUT differ only in that functional group, these energy estimates suggested that MAY’s rhamnosyl-6-C-(4-ketofucosyl) moiety may be crucial to its binding to the NtSI (domain B) active site.

**Figure 4:**
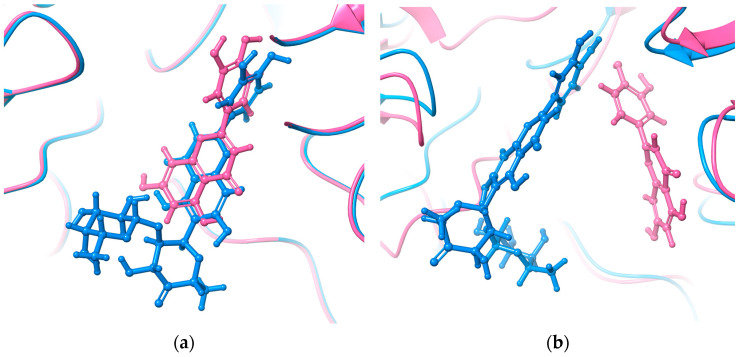
(**a**) Initial docking pose and (**b**) final pose obtained by MD run of MAY (blue) and LUT (pink) in NtSI domain B.

To understand how the rhamnosyl-6-C-(4-ketofucosyl) group affects MAY’s binding pose compared to LUT, the initial docking pose and last MD trajectory snapshot were studied (Figure 4). Their polyphenolic cores are identical. Both molecules were initially in the NtSI (domain B) active site with identical poses (Figure 4a). However, during MD run, MAY’s rhamnosyl-6-C-(4-ketofucosyl) group orientation significantly changed, resulting in a completely different binding mode compared to LUT (Figure 4b).

**Figure 5:**
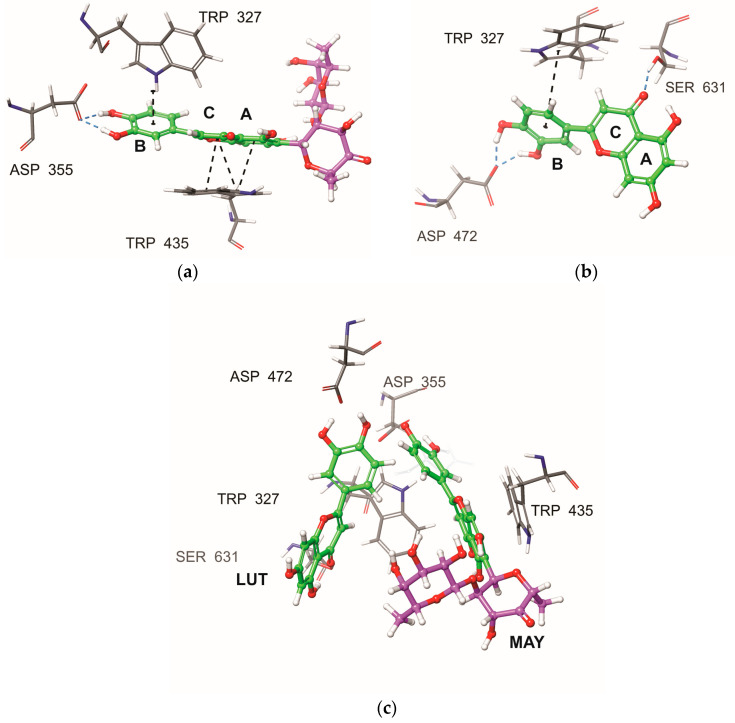
Active site residues of the NtSI domain B that interacted with (**a**) MAY and (**b**) LUT obtained by MD simulation, final screenshot. (**c**) Comparison of binding poses of MAY and LUT. Polyphenolic group: green, rhamnosyl-6-C-(4-ketofucosyl) group: purple, H-bond interaction: blue dashed line, and π–π/aromatic H-bond interaction: black dashed line.

Resulting from the differences in the binding poses of the compounds MAY and LUT, an interaction analysis was conducted (see Table 3 and Figure 5). The analysis indicated that both molecules, MAY and LUT, maintained a strong interaction between the polyphenolic moiety of the B ring and TRP327 amino acid, displaying different types of interactions, for example, with MAY forming an aromatic H-bond and with LUT establishing π–π interactions. Furthermore, distinct interactions were observed, such as the π–π interaction between the A and C rings of the MAY polyphenolic group with the residue TRP435. Additionally, an analysis of formed hydrogen bonds (H-bonds) was performed. Table 3 shows the H-bonds with occupancy values exceeding 15%. It can be observed that the hydroxyl groups of the B ring in the polyphenolic moieties of MAY and LUT formed hydrogen bonds with ASP355 and ASP472, respectively. Finally, a H-bond was observed between the keto group of the C-ring of the polyphenolic moiety of LUT with SER631. These findings clearly indicated that the dissimilar binding poses of both molecules (Figure 5a,b) may be attributed to the influence of the rhamnosyl-6-C-(4-ketofucosyl) group, which pushes the MAY polyphenolic moiety toward the residue TRP327 (Figure 5c), resulting in different interactions with NtSI (domain B) active site residues.

Due to MAY and LUT’s different binding poses, an interaction study was performed (Table 3 and Figure 5). Both MAY and LUT exhibited strong interactions between the polyphenolic moiety of the B ring and TRP327 amino acid, with MAY forming an aromatic H-bond and LUT establishing π–π interactions. Notable interactions include the π–π interactions between the A and C rings of the MAY polyphenolic group and residue TRP435. Additionally, hydrogen bonds (H-bonds) were also analyzed. Table 3 lists H-bonds with occupancy over 15%. The polyphenolic moieties of MAY and LUT established hydrogen bonds with ASP355 and ASP472, respectively, via the B ring’s hydroxyl groups. Finally, the keto group of LUT polyphenolic moiety’s C ring formed a hydrogen bond with SER631. These results showed that the MAY polyphenolic moiety is pushed toward the residue TRP327 (Figure 5c), resulting in different interactions with NtSI (domain B) active site residues, which could explain the different binding poses of both molecules (Figure 5a,b).

**Figure 6:**
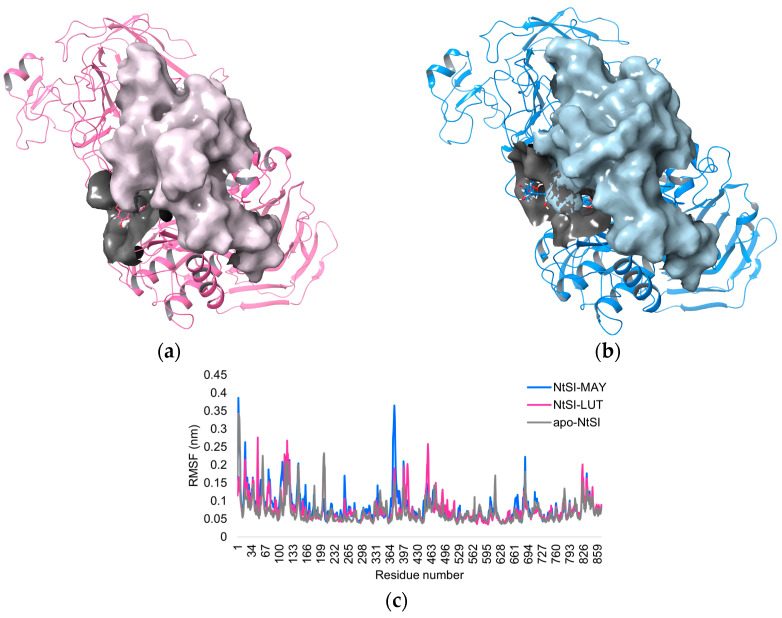
Protein surface of NtSI domain B in complex with (**a**) MAY and (**b**) LUT. (**c**) The RMSF of apo and ligand-bound (MAY and LUT)-NtSI domain B.

In addition, the root mean square fluctuation (RMSF) values were computed for the whole protein structure, illustrating the mean displacement of every backbone atom. The RMSF plot (Figure 6c) shows that in the case of NtSI domain B in complex with MAY (MAY-NtSI), higher fluctuations, around 0.4 nm, were observed in the region comprising residues 360–430 compared to NtSI in apo form and LUT-NtSI. Notably, these residues did not directly interact with MAY, suggesting that this compound may induce long-range effects in the protein, such as allosteric effects, which lead to different changes in the NtSI catalytic domain (Figure 6a,b).

### 2.4. Chemical Reactivity Parameters and Charge Transfer Descriptor

The conceptual density functional theory (DFT)-based chemical reactivity descriptors, such as electronegativity (χ), chemical hardness (η), electrophilicity index (ω), and chemical potential (μ), were defined to gain a deeper understanding of the chemical reactivity of each ligand (ACA, MAY, and LUT) and amino acids of the NtSI (domain B) active site that interacted with the compounds. The values of these chemical reactivity descriptors were estimated using the vertical electron affinity (EA) and the ionization potential (IP). See Table 4 and Table 5. As can be seen in Table 4, the EA values of the ligands fluctuated between 0.23 and 1.95 eV. The highest EA value was obtained for the compound MAY. The greatest IP values were predicted for ACA at 6.16 eV, indicating the possibility of electron loss. Additionally, the chemical hardness (η) of MAY and LUT decreased by approximately 35% and 30%, respectively, compared to ACA. Another chemical reactivity descriptor that was calculated was electronegativity (χ). This descriptor indicates the tendency of a ligand to attract electrons to itself. All ligands shared similar electronegativity values; however, MAY had the most electronegative value (−3.89 eV). Two other important descriptors, namely electrophilicity (ω) and chemical potential (µ), were also estimated. Electrophilicity (ω) represents the ability to accept electrons from the active site of the enzyme, while chemical potential (µ) relates to the ability to attract or transfer electrons. The electrophilicity of ACA was approximately 50% lower than that of MAY and LUT, while MAY exhibited a higher electrophilicity value. Finally, similar chemical potential values were observed for all compounds. However, MAY demonstrated chemical potential values over 17% higher than those of ACA. All of the aforementioned points suggested that MAY could be more effective at interacting with the NtSI (domain B) compared to ACA and LUT.

Considering the importance of the charge transfer in complex formation and oxidative and reductive processes in biological systems, the charge transfer (ΔN) between ACA, MAY, and LUT with amino acids of the NtSI (domain B) active site, which are relevant to ligand binding, were calculated using Equation 8. In this equation, μA and ηA represent the chemical potential and chemical hardness of the ligands, respectively, while μB and ηB correspond to the amino acids of the binding site as determined by MD simulation. The values of μ, η, and ΔN can be observed in Table 5. Values of ΔN < 0 indicate that the charge transfer flows from A to B, where A acts as an electron donor. All molecules, ACA, MAY, and LUT, served as electron donors to the NtSI (domain B). Consequently, the charge transfer in the NtSI (domain B) active site decreases in the following order: MAY > LUT > ACA.

## 3. Discussion

The high costs of diabetes treatment have a significant impact on the public healthcare sector, primarily due to the medical expenses associated with the long-term complications of diabetes [25]. These complications arise from the lack of glycemic control, which has been linked to limitations in available therapies for type 2 diabetes [26,27]. Therefore, it is crucial to explore new therapeutic alternatives for patients with this disease. In this context, natural products, such as maize silks, have emerged as a valuable source of biologically active compounds [10,11,12]. Therefore, the objective of this research was to investigate the potential inhibitory effect of MAY and LUT on the NtSI (domain B) and CtSI (domain D) through MDock, molecular dynamics (MD), and quantum chemical approaches, taking as a reference ACA, a reported antidiabetic drug. LUT is a flavonoid described experimentally as an α-glucosidase inhibitor [14,15,16,17,18], such as the SI enzyme [28].

First, a structural optimization was performed for each compound, ACA, MAY, and LUT. During this optimization, it was observed that the hydroxyl (-OH) groups and rotatable bonds of ACA favored the formation of intramolecular interactions (H-bonds) in the gas phase. However, in a water solution, most of these intramolecular interactions were disrupted. This could be attributed to the competition between the intramolecular H-bonds of -OH groups in ACA and H-bonds formed between these groups and water molecules, resulting in a more linear structure in the aqueous phase. On the other hand, MAY and LUT exhibited similar conformations in both the gas and aqueous phases. This could be due to the lesser formation of intramolecular H-bonds and the rigidity of the polyphenol moiety in these compounds.

Subsequently, only the structure optimized in the aqueous phase was employed for MDock and MD simulations, considering the solvent effect on the compounds, particularly on ACA. The MDock analysis indicated that the binding affinity tends to be higher in NtSI domain B than CtSI domain D. In the same way, these results align with the MD simulation data, where the RMSD values of MAY and LUT did not converge to a stable conformation when complexed with CtSI domain D. This suggests that these molecules may not bind effectively to the CtSI active site. This discrepancy in binding affinity between NtSI and CtSI may be related to the differences in catalytic activities of the SI subunits. NtSI has a substrate specificity for α1–6 isomaltose, while the CtSI subunit specifically acts on the α1–2 linkage of sucrose [29,30]. Eskandari et al. previously observed this difference in inhibition specificity for each subunit [31]. They speculated that CtSI might possess a hydrophobic pocket in the active site that favors the accommodation of hydrophobic groups, thereby prioritizing hydrophobic interactions. Hence, it is suggested that due to the hydrophilic nature of MAY and LUT, and facilitated by the presence of -OH groups, these compounds preferentially bind to NtSI domain B rather than CtSI domain D.

Since the compounds showed a higher binding affinity for NtSI domain B compared to CtSI domain D, a detailed MD analysis was carried out in order to gain further insight into the predicted binding poses obtained for MDock studies of the ligand–NtSI complexes. Additionally, the MD simulations were used to calculate the binding free energy values, considering protein flexibility and system solvation. The MD results revealed that ACA, the positive control, remained stable in both the NtSI (domain B) and CtSI (domain D) active sites. The binding site of ACA observed in this study is consistent with the binding pocket identified by Xiao and collaborators [32].

On the other hand, MAY and LUT showed stable binding conformations in the NtSI (domain B) active site; however, MAY exhibited slight variations in RMSD values. This was primarily due to the rhamnosyl-6-C-(4-ketofucosyl) moiety, which did not remain in a relatively constant position throughout the MD simulation. Patil et al. [33] found a similar pattern of RMSD, where the values increase proportionally with the number of rotatable bonds. This indicates that the structure of MAY displays more flexibility compared to that of LUT, particularly in the rhamnosyl-6-C-(4-ketofucosyl) group, allowing for the generation of a more diverse set of conformations during the extended MD simulation, which leads to higher RMSD values. In contrast, ACA in complex with NtSI domain B did not exhibit significant RMSD fluctuations, despite having more rotatable bonds than LUT. This can be attributed to the stabilization of ACA within the binding pocket through hydrogen bonds formed between ASP472 and ASP571 with the acarvosine core, as well as ASP632 with the maltose core. On the other hand, the rhamnosyl-6-C-(4-ketofucosyl) moiety of MAY was predominantly exposed to the solvent, resulting in higher flexibility and greater RMSD values.

Although the rhamnosyl-6-C-(4-ketofucosyl) substituent of MAY did not directly interact with the protein, it plays a relevant role in enhancing the binding affinity towards the NtSI (domain B) active site. In fact, the binding free energy values of MAY were approximately double those of LUT. Moreover, it has been reported that the glycosylated compounds, such as MAY, are better absorbed by the small intestine than their non-glycosylated variants [34,35,36]. In this context, the binding free energy difference between ACA and MAY (−1.43 Kcal/mol) was lower than that between ACA and LUT (−6.2 Kcal/mol). This suggests that MAY could exhibit better biological activity than LUT. These differences can be attributed to the variations in molecular interactions between MAY and LUT with the target protein.

These molecular interactions were analyzed in more detail by MD simulations. Both molecules, MAY and LUT, as well as ACA, interacted with important residues for NtSI domain B catalysis. MAY and LUT kept the interaction with TRP327. This amino acid is associated with an increased isomaltase activity of NtSI [24,37]. Given that MAY and LUT share an equal polyphenolic core, a similar interaction was expected, but it was found that MAY formed an aromatic hydrogen bond, while LUT engaged in a π–π interaction with TRP327. Other different interactions were observed between these compounds and residues that contribute to NtSI catalytic activity. MAY interacted with ASP355 and TRP435, which are substrate-binding residues, while LUT interacted with ASP472, a residue directly involved in the NtSI catalytic process as a nucleophile [37]. These interactions with important catalytic residues could potentially affect NtSI enzymatic activity. The different binding poses of these ligands can be explained by the influence of the rhamnosyl-6-C-(4-ketofucosyl) moiety of MAY, which pushed the polyphenolic moiety of the molecule towards the residue TRP327. This moiety facilitates interactions with dissimilar catalytic residues, thereby enhancing the binding affinity of MAY for the enzyme. Interestingly, MAY caused more disruption of the NtSI domain B structure than LUT, specifically in the region comprising residues 360–430, which corresponds to a fragment of the catalytic (β/α) 8-barrel domain (residues 270–651) [24,38]. These significant perturbations induced by MAY in this protein fragment suggest that this compound might trigger an allosteric effect in the catalytic (β/α) 8-barrel domain, potentially affecting NtSI enzymatic activity.

The calculated chemical reactivity descriptors provided insights into the chemical behavior of ACA, MAY, and LUT in relation to NtSI domain B. These descriptors, derived from conceptual density functional theory, included electronegativity (χ), chemical hardness (η), electrophilicity index (ω), and chemical potential (μ), which were estimated using the vertical electron affinity (EA) and ionization potential (IP). The results indicated that MAY exhibited a higher electron affinity, suggesting that it can more easily generate an anion compared to LUT and ACA. On the other hand, ACA showed a lower possibility of losing electrons, as indicated by its higher ionization potential (IP). In addition, the chemical hardness values indicated that MAY presented the highest ability to interact with NtSI domain B, indicating its stability and resistance to electron transfer. The electronegativity values suggested that MAY tends to attract electrons, implying a stronger electron-attracting tendency compared to LUT and ACA. The electrophilicity index indicated that MAY presents a high capacity to accept electrons from the NtSI (domain B) active site, demonstrating its ability to act as an electron acceptor in the interaction. Finally, the chemical potential (µ), which represents the tendency of a molecule to attract or transfer electrons, is an important parameter in the calculation of the charge transfer descriptor. In summary, the calculated chemical reactivity descriptors indicated that MAY possesses favorable properties for interacting with the NtSI domain B active site, including a higher electron affinity, stronger electron-attracting tendency, and a greater capacity to accept electrons. These findings contributed to the understanding of the chemical behavior and potential activity of ACA, MAY, and LUT in relation to NtSI.

Considering the high importance of the charge transfer parameter (ΔN) in the formation of complexes, in the oxidative process of biological systems, and as an indicator of specificity and reversibility in many of the biochemical reactions [39], it was calculated between ACA, MAY, and LUT with NtSI domain B using Equation 8 [40]. In this analysis, μA is the chemical potential of the ligands ACA, MAY, and LUT, while μB is the chemical potential for the amino acids of the NtSI (domain B) active site that interacted with the ligands. The chemical hardness ηA and ηB represent the values for ligands and amino acids of the NtSI (domain B) active site, respectively. ΔN represents the fraction of transferred electrons from one system to another. The calculated ΔN values indicate the direction of the charge transfer. ΔN < 0 suggests that the charge transfer flows from A to B, indicating that A acts as an electron donor. Conversely, ΔN > 0 suggests that the charge transfer flows from B to A, indicating that A acts as an electron acceptor. This parameter was used to describe the oxidative damage in DNA bases [41,42]. Also, the charge transfer is a first step to understanding the oxidative damage process in the active site produced by the MAY and LUT ligands, allowing the identification of their behavior and biological activity with the NtSI. Therefore, the results obtained indicated that the ligands MAY and LUT acted as electron acceptors, while the active site amino acids functioned as electron donors. On the other hand, the ACA compound acted as an electron donor, and the amino acids worked as electron acceptors. As a result, the analysis revealed that the oxidative damage in the active site of NtSI domain B showed the following tendency: MAY > LUT > ACA. This coincides with the electrophilicity parameters that indicated that MAY and LUT have the highest capacity to accept electrons from the NtSI (domain B).

The aforementioned findings provide valuable insights into the behavior of the mixed inhibition observed in our previous experimental research, where hydroalcoholic extract polyphenolic compounds from maize silks were tested on a crude α-glucosidases extract [10]. Various polyphenolic molecules can function as competitive or uncompetitive components in mixed inhibition induced by a plant extract. In this case, our results suggested that MAY could be participating as a competitive inhibitor, particularly interacting with the active site of NtSI. The data also provide mechanism insights into the biological activity of MAY and LUT with respect to NtSI, particularly regarding their potential as therapeutic agents against type 2 diabetes. Understanding the charge transfer and oxidative damage processes can aid in subsequent efficacy assessment and the mechanism of action of these ligands in relation to NtSI.

## 4. Methods

### 4.1. Structural Optimization

A global minimum search was performed using an iterative composite procedure approach, which involved methadynamic sampling, regular molecular dynamics sampling, and a genetic structure crossing algorithm. This procedure relied on the extended tight binding GFN*n*-xTB *(n* = 0–2) methods [43,44,45]. This is implemented in the conformer–rotamer ensemble sampling tool (CREST version 2.12) [46]. In the first step, a single geometry for MAY, LUT, and ACA was optimized in water solution using the GFN2-xTB method with the analytical linearized Poisson–Boltzmann (ALPB) model implemented in xtb version 6.5.1. The optimized geometries of MAY and ACA were then used as input for CREST to explore the conformational space of each compound. LUT was excluded from this step as it has fewer rotatable bonds compared to MAY and ACA. The global minimum obtained from CREST, along with selected conformers exhibiting significantly different RMSD values, was further optimized using DFT level of theory. The M06 meta-hybrid functional with the 6-31G(d) basis set was employed for the DFT optimization step [47,48]. This methodology, which has been previously used by our research group, has shown the best correlation with experimental data and statistical analysis [13]. In each molecule, the solvation effect was included through the Solvation Model based on Density (SMD) [49] implicit solvation model. The optimized structures were confirmed to be true local minima calculating the normal vibration modes. Ultrafine grid settings were used, and strict convergence criteria were required for the total energy, set to 10^−8^ atomic units. The geometries were optimized with a Root Mean Square (RMS) forces threshold of 10^−5^ atomic units. DFT calculations were carried out using the Gaussian 16 quantum chemistry software package [50].

### 4.2. Molecular Docking Calculations

The docking molecular simulations of ACA and MAY structures were performed using Mdock in NtSI (domain B) and CtSI (domain D). The initial structure for the NtSI simulation was obtained from the Protein Data Bank (http://www.rcsb.org, accessed on 8 October 2022), PDB-ID: 3LPP [37]. To carry out the Mdock simulations, the monomers NtSI (monomer B) and CtSI (monomer D) were employed along with the ligands ACA, MAY, and LUT. The Mdock calculations were conducted using the AutoDock Vina 1.1.2 [51] software with the Lamarckian genetic algorithm [52]. The water molecules in the enzyme were removed, and only the polar hydrogen atoms were added. The docking area was defined around the active site of domain B, with box dimensions of 50 × 52 × 82 points. The box was centered at x, y, and z coordinates of −39.110, 57.515, and −76.849, respectively. For the domain D active site, the box dimensions were set to 68 × 78 × 78 points, centered at x, y, and z coordinates of 55.897, 96.538, and −8.005, respectively. The grid spacing in both domains was set to 1 Å. All preparation steps were carried out using AutoDock tools 1.5.7 [53].

### 4.3. Molecular Dynamics Simulations

**Figure A2:**
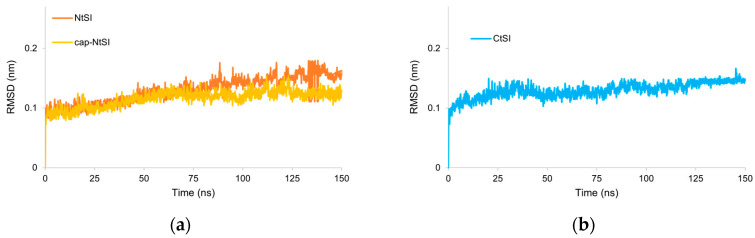
(**a**) RMSD values of cap-NtSI αC atoms compared with cap-free NtSI αC. There was a minimal variation in the RMSD values of cap-NtSI αC atoms compared to cap-free NtSI, the RMSD averages were 0.116 and 0.130 nm, respectively. (**b**) RMSD values of CtSI αC atoms. The coefficient of variation in CtSI domain D RMSD was around 10%.

The docking poses of the ACA, MAY, and LUT molecules in both SI (monomer B and D) with lower negative binding energy values were subjected to MD simulations. A capping group (cap), represented as an acetyl group (ACE), was introduced to the N-terminal domain B in order to achieve a more stable conformation of NtSI (domain B), while a capping group was unnecessary for CtSI (domain D). See more information in Figure A2 in Appendix A. These SI domains were subsequently employed in MD simulations involving the ACA, LUT, and MAY compounds. The MD simulations of the studied complexes were performed using the Chemistry at Harvard Macromolecular Mechanics (CHARMM 36) force field [54] and Nanoscale Molecular Dynamics (NAMD 3) software [55]. Both domains, NtSI and CtSI, were prepared using the CHARMM-GUI server (https://www.charmm-gui.org) [56]. Missing residues and the ACE group, employed to cap the N-terminus, were added, and the PDB file with ID: 3LPP [37] was utilized. The force field parameters for the ligands were generated using the AnteChamber Python Parser InterfacE (ACPYPE 2022.6.6) program [57], which is a Python-based tool for utilizing ANTECHAMBER 22.0 [58], in order to generate topologies for chemical compounds. Geometry optimization and charges were obtained using semiempirical quantum mechanics (SQM) (part of AmberTools 23 [59]) with Self Consistent charge Density-functional Tight-binding (SCC-DFTB) Hamiltonian [48] to construct the ligands parameters. The principal axes of the protein were aligned with the coordinate axes. Then, the protein–ligand systems were solvated using the Transferable Intermolecular Potential with 3 points (TIP3P) water model in a box with periodic boundary conditions, extending 15 Angstrom in each direction. To neutralize the charge, Na+ and Cl− ions were added at a concentration of 0.15 mM using Visual Molecular Dynamics (VMD 1.9.3) [60]. Energy minimization was performed using the conjugate gradient algorithm for 10,000 steps. After minimization, the systems were heated to 310 K during 500 ps in the constant-temperature, constant-volume (NVT) ensemble. Finally, the systems were maintained at 310 K and 1 atm for 150 ns using the Langevin piston Nose–Hoover method [61,62]. The Smooth Particle Mesh Ewald (PME) protocol was used for handling long-range electrostatic interactions [63]. Visualization and analysis of the simulation were performed using VMD, including evaluation of RMSD and the H-bond analysis.

### 4.4. Binding Free Energy Calculations

In the present study, the Molecular Mechanics Poisson–Boltzmann Surface Area (MM-PBSA) method was employed to calculate the binding free energy. This method, originally developed by Kollman [64], allows for the estimation of the binding free energy between a protein and a ligand using the following equation:

(1) $$G_{bind}=G_{PL}-G_{P}-G_{L}$$

where G_PL_, G_P_, and G_L_ are the free energies of the protein–ligand complex, protein, and ligand, respectively. The free energy of each component is estimated by the following sum:

(2) $$G=\langle E_{MM}\rangle +\langle G_{solv}\rangle +\langle G_{np}\rangle -T\langle S_{MM}\rangle$$

The first term is the standard MM energy, which represents the sum of the internal energy of the molecule, including bonded terms, the electrostatics, and van der Waals interactions. The second term is the polar solvation energy of the molecule, obtained from the solution of the Poisson−Boltzmann (PB) equation (in this study, calculated using the Adaptative Poisson-Boltzmann Solver (APBS 3.0) [65] package). The third term is the nonpolar solvation energy, estimated from the solvent-accessible surface area of the molecule (calculated using VMD). The last term consists of the temperature T and the entropy contribution, usually obtained from a normal-mode calculation of the harmonic frequencies at the MM level. In this study, the last term was ignored due to computational expense and the significant uncertainty it introduces in the final results [66]. The terms in Equation 2 represent averages of energies obtained from a specific number of molecular dynamics snapshots (500 in this study). For practical purposes, all the terms were calculated from a single MD simulation corresponding to the protein–ligand complex. Finally, the total binding free energy was calculated using the Calculation of Free Energy (CaFE 1.0) [67] VMD plugin.

### 4.5. Chemical Reactivity and Charge Transfer Calculations

The chemical reactivity descriptors of the molecular systems studied, including ACA, MAY, LUT, and amino acids of NtSI domain B, were calculated using the DFT conceptual framework. These parameters, such as ionization potential (IP) [68,69], electron affinity (EA) [68,69], chemical harness (η) [70], chemical potential (µ), electrophilicity (ω) [71], and the charger transfer (ΔN) [40,72], were determined by applying the following equations:

(3) $$IP=E_{\left(N-1\right)}-E_{\left(N\right)}$$

(4) $$EA=E_{\left(N\right)}-E_{\left(N+1\right)}$$

(5) $$\eta =\frac{1}{2}\left(IP-EA\right)$$

(6) $$\chi =-\mu =\frac{1}{2}\left(IP+EA\right)$$

(7) $$\omega =\frac{\mu^{2}}{2\eta }$$

(8) $$\Delta N=\frac{\mu_{B}-\mu_{A}}{2\left(\eta_{A}+\eta_{B}\right)}$$

The IP and EA were calculated by the energy difference E_(N−1)_ − E_(N)_ and E_(N)_ − E_(N+1)_, respectively. In these equations, the energy (E) is considered as a function of the number of electrons (N). The η and µ were used to determine ΔN, which describes the fractional number of transferred electrons from system A to system B or vice versa. System A corresponds to ACA, MAY, and LUT, while system B corresponds to any of the amino acids in the active site of NtSI domain B. A positive value of ΔN indicates that the ligands act as an electron acceptor, while a negative value of ΔN indicates that the ligands act as an electron donor.

## 5. Conclusions

In this study, a theoretical analysis using molecular docking, molecular dynamics, and quantum chemical calculations was conducted to investigate two polyphenolic compounds derived from maize silks, maysin and luteolin, along with the drug acarbose, which is used to treat type 2 diabetes mellitus. This analysis allowed a complete study of the molecular interactions of these compounds with sucrase-isomaltase (SI), an enzyme that presents two duplicated catalytic domains: the N-terminal (NtMGAM, NtSI) and C-terminal (CtMGAM, CtSI).

The obtained results indicated that the polyphenolic compounds, maysin and luteolin, could inhibit the enzymatic activity of SI by preferentially binding to the NtSI, as revealed by molecular docking and molecular dynamics analysis. In this regard, maysin emerged as the most active flavonoid, displaying a binding free energy similar to that of acarbose. This increase in the binding affinity of maysin with NtSI, compared to luteolin, can be attributed to the presence of the rhamnosyl-6-C-(4-ketofucosyl) moiety in its structure. Furthermore, the chemical hardness value of maysin indicated that this compound might interact more easily with the active site of NtSI than acarbose and luteolin. Additionally, the charge transfer descriptor indicated that both maysin and luteolin exhibited a higher ability to interact and induce oxidative damage at the NtSI active site (where 100% and 89% of the active site amino acids showed oxidation in the presence of each compound, respectively). These oxidative damage processes could be related to the mechanism of action of these ligands with the amino acids of the NtSI active site.

In conclusion, this work significantly contributes to the investigation of flavonoid-mediated inhibition of human SI, providing a better understanding of the chemical features and potential inhibitory effects of maysin and luteolin, particularly targeting the NtSI active site and inducing oxidative damage at its catalytic site. These predictions can also help to interpret the existing results or predict future experimental results. Moreover, the data obtained in this study are important for hypothesis generation and experimental design to further assess the therapeutic potential of these compounds as possible therapies against type 2 diabetes.

## Figures and Tables

**Table 1:** Difference in relative energies (Kcal/mol) of global minimum between other conformers of ACA and MAY. NA: not applicable.

Conformer	Difference in Relative Energy and Structure	
	ACA	MAY
Global minimum	0.0 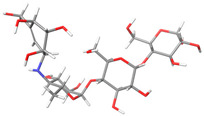	0.0 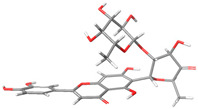
Conformer 1	2.8 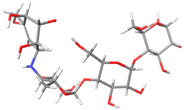	3.5 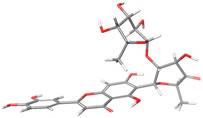
Conformer 2	7.1 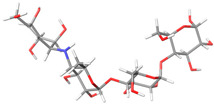	5.0 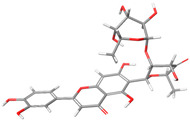
Conformer 3	8.4 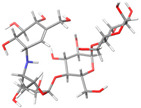	7.4 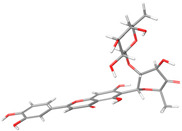
Conformer 4	NA	9.7 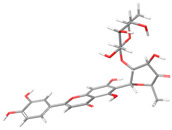

**Table 2:** Binding free energy calculated for each complex NtSI (domain B) and CtSI (domain D), PDB-ID: 3LPP, with the compounds ACA, MAY, and LUT and molecular interaction analysis predicted by MDock calculations. The residues that interacted with each ligand are highlighted in bold type.

Ligand	Domain	Binding Free Energy (Kcal/mol)	Binding Site Residues
ACA	B	−7.8	TRP327, ASN328, LYS330, ILE356, **TRP435 (H-bond)**, TRP470, **ASP472 (H-bond)**, MET473, ARG555, **ASP571 (H-bond)**, PHE604, HIS629, and ASP632
	D	−8.8	**ASN43 (H-bond)**, ILE45, PRO46, GLU47, GLN48, **PHE49 (H-bond)**, PRO50, SER68, LEU69, THR224, PHE272, ARG282, and LYS594
MAY	B	−8.0	ASP231, LEU233, TRP327, **ASP355 (H-bond)**, ILE356, **TRP435 (π–π interaction)**, TRP470, ASP472, MET473, SER477, PHE479, **LYS509 (H-bond)**, **HIS629 (H-bond)**, and SER631
	D	−7.5	ASP231, GLN232, LEU233, TRP327, **ASP355 (H-bond)**, ILE392, TRP435, TRP470, and LYS509
LUT	B	−8.6	**ASP231 (H-bond)**, TRP327, **ASP355 (H-bond)**, ILE392, TRP435, TRP470, ASP472, PHE479, **LYS509 (H-bond)**, ASP571, PHE600, and SER631
	D	−7.4	LEU311, ALA313, ARG549, ARG563, HIS629, and **LYS805 (π–π interaction)**

**Table 3:** Analysis of H-bonds (occupancy > 15%), π–π interaction, and aromatic H-bond formed between NtSI (domain B) with ACA, MAY, and LUT during MD simulation. NA: not applicable.

Ligand	Molecular Interactions		
	H-Bonds (Occupancy in %)	π–π Interaction	Aromatic H-Bond
ACA	ASP472 (55.43), ASP571 (40.72) ASP632 (15.17)	NA	NA
MAY	ASP355 (87.89)	TRP435	TRP327
LUT	ASP472 (88.61), SER631 (17.29)	TRP327	NA

**Table 4:** Reactivity parameters of docked ligands in NtSI domain B, calculated by M06/6-31G(d) level of theory. Electron affinity (EA), ionization potential (IP), chemical hardness (η), electronegativity (χ), electrophilicity (ω), and chemical potential (µ). All values are presented in eV.

Ligand	EA	IP	η	χ	ω	μ
ACA	0.01	6.12	3.05	3.07	1.54	−3.07
MAY	1.95	5.83	1.94	3.89	3.90	−3.89
LUT	1.74	5.88	2.07	3.81	3.50	−3.81

**Table 5:** Reactivity parameters and charge transfer descriptor calculated for amino acids of the NtSI domain B, interacting with ACA, MAY, and LUT. Chemical hardness (η), chemical potential (µ), and charge transfer (ΔN). The values of η and µ are presented in eV.

Ligand	Residue Active Site	η	µ	ΔN
ACA	TRP327-ASN328	2.15	−3.23	−0.016
	LYS330	3.06	−3.68	−0.050
	ILE356	2.97	−3.52	−0.037
	TRP435	2.24	−3.08	−0.001
	TRP470	2.25	−2.87	0.018
	ASP472-MET473	2.73	−3.27	−0.018
	TRP568	2.27	−3.04	0.002
	ASP571	2.72	−3.21	−0.013
	PHE604	2.80	−3.45	−0.033
	ASP632	2.67	−3.33	−0.023
MAY	ASP231	2.71	−3.41	0.052
	TRP327-ASN328	2.07	−3.09	0.100
	ASP355-ILE356-ASP357	2.83	−3.03	0.090
	ILE392	3.07	−3.55	0.034
	TRP435	2.28	−3.03	0.102
	MET473	2.52	−3.14	0.084
	PHE479	2.80	−3.53	0.038
	LYS509	3.01	−3.65	0.024
LUT	LEU233	3.04	−3.65	0.016
	TRP327	2.30	−3.12	0.078
	ILE356	3.06	−3.44	0.036
	ILE392	3.14	−3.54	0.026
	TRP470	2.19	−3.04	0.090
	ASP472-MET473	2.64	−3.25	0.060
	ASP571	2.76	−3.10	0.074
	PHE604-VAL605	2.70	−3.45	0.037
	SER631	2.96	−3.89	−0.008

## Data Availability

Not applicable.

## Appendix A

**Table A1:** Binding free energy contributions for ACA-, LUT-, and MAY-NtSI domain B complexes calculated using the MM/PBSA method. All energies are in Kcal/mol. Van der Waals interaction energies (vdW), electrostatic energy (elec), polar contribution (PB), nonpolar contribution (SA), gas-phase contribution (Ggas), solvent contribution (Gsol), and total binding free energy (Gbind).

ID Compound	vdW	elec	PB	SA	Ggas	Gsol	Gbind
ACA	−17.42	−105.35	110.22	−2.97	−122.77	107.25	−15.52
MAY	−27.61	−58.28	75.05	−3.26	−85.89	71.79	−14.09
LUT	−18.85	−53.90	66.27	−2.85	−72.75	63.43	−9.32

## References

[1] Preedy V.R., Watson R.R.R. (2010). Handbook of Disease Burdens and Quality of Life Measures.

[2] Rother K.I. (2007). Diabetes Treatment—Bridging the Divide. N. Engl. J. Med..

[3] Cipriani-Thorne E., Quintanilla A. (2010). Diabetes Mellitus Tipo 2 y Resistencia a La Insulina. Rev. Medica Hered..

[4] Nair S.S., Kavrekar V., Mishra A. (2013). In Vitro Studies on Alpha Amylase and Alpha Glucosidase Inhibitory Activities of Selected Plant Extracts. Eur. J. Exp. Biol..

[5] Lin A.H.-M., Lee B.-H., Chang W.-J. (2016). Small Intestine Mucosal α-Glucosidase: A Missing Feature of in Vitro Starch Digestibility. Food Hydrocoll..

[6] Ernst H.A., Leggio L.L., Willemoës M., Leonard G., Blum P., Larsen S. (2006). Structure of the Sulfolobus Solfataricus α-Glucosidase: Implications for Domain Conservation and Substrate Recognition in GH31. J. Mol. Biol..

[7] Gericke B., Schecker N., Amiri M., Naim H.Y. (2017). Structure-Function Analysis of Human Sucrase-Isomaltase Identifies Key Residues Required for Catalytic Activity. J. Biol. Chem..

[8] Rose D.R., Chaudet M.M., Jones K. (2018). Structural Studies of the Intestinal α-Glucosidases, Maltase-Glucoamylase and Sucrase-Isomaltase. J. Pediatr. Gastroenterol. Nutr..

[9] Clissold S.P., Edwards C. (1988). Acarbose: A Preliminary Review of Its Pharmacodynamic and Pharmacokinetic Properties, and Therapeutic Potential. Drugs.

[10] Alvarado-Díaz C.S., Gutiérrez-Méndez N., Mendoza-López M.L., Rodríguez-Rodríguez M.Z., Quintero-Ramos A., Landeros-Martínez L.L., Rodríguez-Valdez L.M., Rodríguez-Figueroa J.C., Pérez-Vega S., Salmeron-Ochoa I. (2019). Inhibitory Effect of Saccharides and Phenolic Compounds from Maize Silks on Intestinal α-Glucosidases. J. Food Biochem..

[11] Sabiu S., O’neill F.H., Ashafa A.O.T. (2016). Kinetics of α-Amylase and α-Glucosidase Inhibitory Potential of *Zea Mays* Linnaeus (Poaceae), Stigma Maydis Aqueous Extract: An in Vitro Assessment. J. Ethnopharmacol..

[12] Wang K.-J., Zhao J.-L. (2019). Corn Silk (*Zea Mays* L.), a Source of Natural Antioxidants with α-Amylase, α-Glucosidase, Advanced Glycation and Diabetic Nephropathy Inhibitory Activities. Biomed. Pharmacother..

[13] Landeros-Martínez L.-L., Gutiérrez-Méndez N., Palomares-Báez J.P., Sánchez-Bojorge N.-A., Flores-De los Ríos J.P., Piñón-Castillo H.A., Chávez-Rojo M.A., Rodriguez-Valdez L.-M. (2021). The Oxidative Process of Acarbose, Maysin, and Luteolin with Maltase-Glucoamylase: Molecular Docking and Molecular Dynamics Study. Appl. Sci..

[14] Lim J., Zhang X., Ferruzzi M.G., Hamaker B.R. (2019). Starch Digested Product Analysis by HPAEC Reveals Structural Specificity of Flavonoids in the Inhibition of Mammalian α-Amylase and α-Glucosidases. Food Chem..

[15] Yan J., Zhang G., Pan J., Wang Y. (2014). α-Glucosidase Inhibition by Luteolin: Kinetics, Interaction and Molecular Docking. Int. J. Biol. Macromol..

[16] Djeujo F.M., Ragazzi E., Urettini M., Sauro B., Cichero E., Tonelli M., Froldi G. (2022). Magnolol and Luteolin Inhibition of α-Glucosidase Activity: Kinetics and Type of Interaction Detected by in Vitro and in Silico Studies. Pharmaceuticals.

[17] Djeujo F.M., Stablum V., Pangrazzi E., Ragazzi E., Froldi G. (2023). Luteolin and Vernodalol as Bioactive Compounds of Leaf and Root Vernonia Amygdalina Extracts: Effects on α-Glucosidase, Glycation, ROS, Cell Viability, and in Silico ADMET Parameters. Pharmaceutics.

[18] Vonia S., Hartati R., Insanu M. (2022). In Vitro Alpha-Glucosidase Inhibitory Activity and the Isolation of Luteolin from the Flower of Gymnanthemum Amygdalinum (Delile) Sch. Bip Ex Walp. Molecules.

[19] Stefaniu A. (2019). Introductory Chapter: Molecular Docking and Molecular Dynamics Techniques to Achieve Rational Drug Design. Molecular Docking and Molecular Dynamics.

[20] Rozhenko A.B. (2014). Density Functional Theory Calculations of Enzyme–Inhibitor Interactions in Medicinal Chemistry and Drug Design. Application of Computational Techniques in Pharmacy and Medicine.

[21] Merugu R., Neerudu U.K., Dasa K., Singh K.V. (2016). Molecular Docking Studies of Deacetylbisacodyl with Intestinal Sucrase-Maltase Enzyme. Int. J. Adv. Sci. Res..

[22] Heidari A. (2016). Molecular Dynamics and Monte–Carlo Simulations for Replacement Sugars in Insulin Resistance, Obesity, LDL Cholesterol, Triglycerides, Metabolic Syndrome, Type 2 Diabetes and Cardiovascular Disease: A Glycobiological Study. J. Glycobiol..

[23] Luo F., Gao J., Cheng Y.-H., Cui W., Ji M.-J. (2012). Interaction Mechanisms of Inhibitors of Glucoamylase by Molecular Dynamics Simulations and Free Energy Calculations. Acta Phys. Chim. Sin..

[24] Sim L., Quezada-Calvillo R., Sterchi E.E., Nichols B.L., Rose D.R. (2008). Human Intestinal Maltase–Glucoamylase: Crystal Structure of the N-Terminal Catalytic Subunit and Basis of Inhibition and Substrate Specificity. J. Mol. Biol..

[25] Arredondo A., Reyes G. (2013). Health Disparities from Economic Burden of Diabetes in Middle-Income Countries: Evidence from México. PLoS ONE.

[26] Adler A.I., Stratton I.M., Neil H.A.W., Yudkin J.S., Matthews D.R., Cull C.A., Wright A.D., Turner R.C., Holman R.R. (2000). Association of Systolic Blood Pressure with Macrovascular and Microvascular Complications of Type 2 Diabetes (UKPDS 36): Prospective Observational Study. BMJ.

[27] The Action to Control Cardiovascular Risk in Diabetes Study Group (2008). Effects of Intensive Glucose Lowering in Type 2 Diabetes. N. Engl. J. Med..

[28] Proença C., Rufino A.T., de Oliveira J.M.P.F., Freitas M., Fernandes P.A., Silva A.M., Fernandes E. (2022). Inhibitory Activity of Flavonoids against Human Sucrase-Isomaltase (α-Glucosidase) Activity in a Caco-2/TC7 Cellular Model. Food Funct..

[29] Lee B.-H., Lin A.H.-M., Nichols B.L., Jones K., Rose D.R., Quezada-Calvillo R., Hamaker B.R. (2014). Mucosal C-Terminal Maltase-Glucoamylase Hydrolyzes Large Size Starch Digestion Products That May Contribute to Rapid Postprandial Glucose Generation. Mol. Nutr. Food Res..

[30] Lim J., Kim D.K., Shin H., Hamaker B.R., Lee B.-H. (2019). Different Inhibition Properties of Catechins on the Individual Subunits of Mucosal α-Glucosidases as Measured by Partially-Purified Rat Intestinal Extract. Food Funct..

[31] Eskandari R., Jones K., Rose D.R., Pinto B.M. (2011). Selectivity of 3′-O-Methylponkoranol for Inhibition of N-and C-Terminal Maltase Glucoamylase and Sucrase Isomaltase, Potential Therapeutics for Digestive Disorders or Their Sequelae. Bioorg. Med. Chem. Lett..

[32] Li X., Qian K., Han W. (2021). Prediction of Hyaluronic Acid Target on Sucrase-Isomaltase (SI) with Reverse Docking and Molecular Dynamics Simulations for Inhibitors Binding to SI. PLoS ONE.

[33] Patil R., Chikhale R., Khanal P., Gurav N., Ayyanar M., Sinha S., Prasad S., Dey Y.N., Wanjari M., Gurav S.S. (2021). Computational and Network Pharmacology Analysis of Bioflavonoids as Possible Natural Antiviral Compounds in COVID-19. Inform. Med. Unlocked.

[34] Holick M., Ramanathan H. (2001). Glycosides and Orthoester Glycosides of Glucocorticoids and Uses Thereof. U.S. Patent Application.

[35] Holick M.F., Ramanathan H. (2007). Glycuronamides, Glycosides and Orthoester Glycosides of Fluoxetine, Analogs and Uses Thereof. U.S. Patent.

[36] MacCormick S., Veeneman G.H. (2023). Method for Improving the Oral Bioavailability of a Drug. U.S. Patent.

[37] Sim L., Willemsma C., Mohan S., Naim H.Y., Pinto B.M., Rose D.R. (2010). Structural Basis for Substrate Selectivity in Human Maltase-Glucoamylase and Sucrase-Isomaltase N-Terminal Domains. J. Biol. Chem..

[38] Nichols B.L., Avery S., Sen P., Swallow D.M., Hahn D., Sterchi E. (2003). The Maltase-Glucoamylase Gene: Common Ancestry to Sucrase-Isomaltase with Complementary Starch Digestion Activities. Proc. Natl. Acad. Sci. USA.

[39] Landeros-Martinez L.-L., Glossman-Mitnik D., Orrantia-Borunda E., Flores-Holguin N. (2018). A Combined Molecular Docking and Electronic Structure Study for a Breast Cancer Drug Design. Molecular Docking.

[40] Padmanabhan J., Parthasarathi R., Subramanian V., Chattaraj P.K. (2007). Electrophilicity-Based Charge Transfer Descriptor. J. Phys. Chem. A.

[41] Wan C., Fiebig T., Schiemann O., Barton J.K., Zewail A.H. (2000). Femtosecond Direct Observation of Charge Transfer between Bases in DNA. Proc. Natl. Acad. Sci. USA.

[42] Kanvah S., Schuster G.B. (2005). The Sacrificial Role of Easily Oxidizable Sites in the Protection of DNA from Damage. Nucleic Acids Res..

[43] Bannwarth C., Ehlert S., Grimme S. (2019). GFN2-xTB—An Accurate and Broadly Parametrized Self-Consistent Tight-Binding Quantum Chemical Method with Multipole Electrostatics and Density-Dependent Dispersion Contributions. J. Chem. Theory Comput..

[44] Grimme S., Bannwarth C., Shushkov P. (2017). A Robust and Accurate Tight-Binding Quantum Chemical Method for Structures, Vibrational Frequencies, and Noncovalent Interactions of Large Molecular Systems Parametrized for All Spd-Block Elements (Z = 1–86). J. Chem. Theory Comput..

[45] Bannwarth C., Caldeweyher E., Ehlert S., Hansen A., Pracht P., Seibert J., Spicher S., Grimme S. (2021). Extended Tight-Binding Quantum Chemistry Methods. Wiley Interdiscip. Rev. Comput. Mol. Sci..

[46] Pracht P., Bohle F., Grimme S. (2020). Automated Exploration of the Low-Energy Chemical Space with Fast Quantum Chemical Methods. Phys. Chem. Chem. Phys..

[47] Hariharan P.C., Pople J.A. (1973). The Influence of Polarization Functions on Molecular Orbital Hydrogenation Energies. Theor. Chim. Acta.

[48] Francl M.M., Pietro W.J., Hehre W.J., Binkley J.S., Gordon M.S., DeFrees D.J., Pople J.A. (1982). Self-Consistent Molecular Orbital Methods. XXIII. A Polarization-Type Basis Set for Second-Row Elements. J. Chem. Phys..

[49] Marenich A.V., Cramer C.J., Truhlar D.G. (2009). Universal Solvation Model Based on Solute Electron Density and on a Continuum Model of the Solvent Defined by the Bulk Dielectric Constant and Atomic Surface Tensions. J. Phys. Chem. B.

[50] Frisch M.J., Trucks G.W., Schlegel H.B., Scuseria G.E., Robb M.A., Cheeseman J.R., Scalmani G., Barone V., Mennucci B., Petersson G.A. (2016). Gaussian16.

[51] Trott O., Olson A.J. (2010). AutoDock Vina: Improving the Speed and Accuracy of Docking with a New Scoring Function, Efficient Optimization, and Multithreading. J. Comput. Chem..

[52] Morris G.M., Goodsell D.S., Halliday R.S., Huey R., Hart W.E., Belew R.K., Olson A.J. (1998). Automated Docking Using a Lamarckian Genetic Algorithm and an Empirical Binding Free Energy Function. J. Comput. Chem..

[53] Huey R., Morris G.M., Forli S. (2012). Using AutoDock 4 and AutoDock Vina with AutoDockTools: A Tutorial. Scripps Res. Inst. Mol. Graph. Lab..

[54] Vanommeslaeghe K., Hatcher E., Acharya C., Kundu S., Zhong S., Shim J., Darian E., Guvench O., Lopes P., Vorobyov I. (2010). CHARMM General Force Field: A Force Field for Drug-like Molecules Compatible with the CHARMM All-Atom Additive Biological Force Fields. J. Comput. Chem..

[55] Phillips J.C., Braun R., Wang W., Gumbart J., Tajkhorshid E., Villa E., Chipot C., Skeel R.D., Kale L., Schulten K. (2005). Scalable Molecular Dynamics with NAMD. J. Comput. Chem..

[56] Jo S., Kim T., Iyer V.G., Im W. (2008). CHARMM-GUI: A Web-Based Graphical User Interface for CHARMM. J. Comput. Chem..

[57] Sousa da Silva A.W., Vranken W.F. (2012). ACPYPE-Antechamber Python Parser Interface. BMC Res. Notes.

[58] Wang J., Wang W., Kollman P.A., Case D.A. (2001). Antechamber: An Accessory Software Package for Molecular Mechanical Calculations. J. Am. Chem. Soc..

[59] Case D.A., Aktulga H.M., Belfon K., Ben-Shalom I.Y., Berryman J.T., Brozell S.R., Cerutti D.S., Cheatham T.E., Cisneros G.A., Cruzeiro V.W.D. (2022). Amber 2022.

[60] Humphrey W., Dalke A., Schulten K. (1996). VMD: Visual Molecular Dynamics. J. Mol. Graph..

[61] Martyna G.J., Tobias D.J., Klein M.L. (1994). Constant Pressure Molecular Dynamics Algorithms. J. Chem. Phys..

[62] Feller S.E., Zhang Y., Pastor R.W., Brooks B.R. (1995). Constant Pressure Molecular Dynamics Simulation: The Langevin Piston Method. J. Chem. Phys..

[63] Deniz U., Ozkirimli E., Ulgen K.O. (2016). A Systematic Methodology for Large Scale Compound Screening: A Case Study on the Discovery of Novel S1PL Inhibitors. J. Mol. Graph. Model..

[64] Kollman P.A., Massova I., Reyes C., Kuhn B., Huo S., Chong L., Lee M., Lee T., Duan Y., Wang W. (2000). Calculating Structures and Free Energies of Complex Molecules: Combining Molecular Mechanics and Continuum Models. Acc. Chem. Res..

[65] Baker N.A., Sept D., Joseph S., Holst M.J., McCammon J.A. (2001). Electrostatics of Nanosystems: Application to Microtubules and the Ribosome. Proc. Natl. Acad. Sci. USA.

[66] Hou T., Wang J., Li Y., Wang W. (2011). Assessing the Performance of the MM/PBSA and MM/GBSA Methods. 1. The Accuracy of Binding Free Energy Calculations Based on Molecular Dynamics Simulations. J. Chem. Inf. Model..

[67] Liu H., Hou T. (2016). CaFE: A Tool for Binding Affinity Prediction Using End-Point Free Energy Methods. Bioinformatics.

[68] Foresman J.B., Frisch A. (1996). Exploring Chemistry with Electronic Structure Methods.

[69] Berger R. (2004). Computational Chemistry. Introduction to the Theory and Applications of Molecular and Quantum Mechanics. Von Errol G. Lewars. Angew. Chem. Int. Ed..

[70] Parr R.G., Pearson R.G. (1983). Absolute Hardness: Companion Parameter to Absolute Electronegativity. J. Am. Chem. Soc..

[71] Parr R.G., Szentpály L.V., Liu S. (1999). Electrophilicity Index. J. Am. Chem. Soc..

[72] Wang X., Song L., Tian C., He J., Wang S., Wang J., Li C. (2017). DFT Investigation of the Effects of Coexisting Cations and Complexing Reagents on Ni (II) Adsorption by a Polyvinylidene Fluoride-Type Chelating Membrane Bearing Poly (Amino Phosphonic Acid) Groups. Metals.

